# Targeted glypican-3 gene transcription inhibited the proliferation of human hepatoma cells by specific short hairpin RNA

**DOI:** 10.1007/s13277-012-0593-y

**Published:** 2012-11-29

**Authors:** Dandan Yu, Zhizhen Dong, Min Yao, Wei Wu, Meijuan Yan, Xiaodi Yan, Liwei Qiu, Jie Chen, Wenli Sai, Dengfu Yao

**Affiliations:** 1 Research Center of Clinical Medicine, Affiliated Hospital of Nantong University, 20 West Temple Road, Nantong, 226001 Jiangsu Province China; 2 Department of Diagnostics, Medical School of Nantong University, 19 Qixiu Road, Nantong, 226001 Jiangsu Province China; 3 Medical School, Nantong University, 19 Qixiu Road, Nantong, 226001 Jiangsu Province China; 4 Institute of Clinical Oncology, Affiliated Hospital of Nantong University, 20 West Temple Road, Nantong, 226001 Jiangsu Province China

**Keywords:** Hepatocellular carcinoma, Glypican-3, shRNA, Transfection, Gene silencing

## Abstract

Hepatocellular carcinoma (HCC) is a highly chemoresistant cancer with no effective systemic therapy. Despite of surgical or locoregional therapies, prognosis remains poor because of high tumor recurrence or progression, and currently, there are no well-established effective adjuvant therapies. Glypican-3 (GPC-3) is specifically overexpressed in hepatoma and perhaps is a valuable molecular target for HCC therapy. In this present study, the effect of silencing GPC-3 gene transcription on human HepG2 cell proliferation was investigated by constructing GPC-3 short hairpin RNA (shRNA) plasmid. After HepG2 cells were transfected with the most efficient shRNA, GPC-3 mRNA expression (90.4 %) was inhibited significantly and estimated by fluorescence quantitative reverse transcriptase-polymerase chain reaction, and the result was accordance with downregulation at the protein level. The percentage of the cell proliferation was down to 28.9 % in the shRNA group and 19.9 % in the shRNA plus sorafenib group. The cell cycles were arrested in the G_1_ phase (65.6 %) and the apoptosis rate was increasing (66.75 %) in the shRNA1 group with significant alteration compared with that in the negative-shRNA group. Specific shRNA might intervene effectively GPC-3 activation and inhibit tumor cell proliferation, suggesting that GPC-3 gene should be a potential molecular target for HCC therapy.

## Introduction

Hepatocellular carcinoma (HCC) is one of the most common malignancies worldwide and its incidence is still increasing [1, 2]. While the primary curative treatment for HCC is surgical resection, a major obstacle for its treatment is the high frequency of tumor recurrence even after curative resection [3, 4]. Approximately 85 % of HCCs are not candidates for curative treatments at the time of diagnosis; hence, palliative modalities (transcatheter arterial chemoembolization, radiofrequency ablation, systemic chemotherapy) are used [5, 6]. An increasing knowledge in molecular biology of HCC will increase the possibilities of targeted therapy [7, 8]. Molecularly targeted therapies have recently expanded the options available for advanced HCC [4, 9, 10]. The inhibitions of mTOR (rapamycin), VEGF (sunitinib), EGF (erlotinib), and multi-tyrosine kinases (sorafenib) are promising [11]. Alteration of the Wnt pathway, retinoid compounds, inhibition of cell cycle as well as the proteosome, and epigenetic therapy can be other potential promising targets for HCC [12].

Glypican (GPC) is a family of heparan sulfate proteoglycans that are bound to the cell surface by a lipid anchor [13]. Members (GPC-1–6) of this family have been identified in mammals. Glypican-3 (GPC-3) is located at Xq26.1 area on chromosome and promotes hepatoma cell growth by stimulating canonical Wnt signaling. There is increasing evidence indicating that the structural requirements for GPC-3 activation are cell-type specific, and its core protein is processed by a furin-like convertase [14, 15]. Recent studies have revealed that GPC-3 is specifically overexpressed in HCC tissues, and oncofetal antigen glypican-3 has been a promising early diagnostic marker for HCC diagnosis and prognosis [16–18]. However, the effect of the downregulation of GPC-3 expression on hepatoma cell proliferation was still not clearly defined. In this present study, a specific short hairpin RNA (shRNA) was screened and applied to silence GPC-3 gene transcription for observing its effect on proliferation of human hepatoma cells.

## Materials and methods

### Cell culture

Human HepG2 cell lines, obtained from the Chinese Academy of Sciences (Shanghai, China), show a high expression of GPC-3, which is in contrast to its parental tumor. Cells were cultured in high glucose Dulbecco’s modified Eagle’s medium (DMEM; Gibco BRL, USA) supplemented with 10 % heat-inactivated fetal bovine serum (Gibco BRL, USA) in a 5 % CO_2_-humidified atmosphere at 37 °C.

### Plasmid constructs and transfection

Four pairs of GPC-3-shRNA targeting different sites of GPC-3 gene were designed according to human GPC-3 sequence (GenBank: BC035972), synthesized, and then constructed (GenePharma Co., Ltd., Shanghai, China; Table 1). After annealing and primer extension, shRNAs were inserted into pGPU6/GFP/Neo vector and transformed into *Escherichia coli* for screening. The pGPU6/GFP/Neo-negative control vector contains a shRNA sequence that does not suppress the expression of human GPC-3 gene. All the inserted sequences were confirmed by BamH I and Pst I digestion and sequencing. The transfection reagent GenJet^TM^ DNA In Vitro Transfection Reagent for HepG2 Cells (Ver. II; SignaGen, Gaithersburg, MD, USA) was used to transfect shRNA plasmid vector into HepG2 cells according to the manufacturer’s protocol. In brief, HepG2 cells were plated in six wells at 18 to 24 h prior to transfection so that the monolayer cell density reached to 60–70 % confluency for use.

**Table 1:** Oligonucleotide sequences of GPC-3 shRNA in pGPU6/GFP/Neo vector

Name	Sense/antisense strands of shRNA (5′→3′)
GPC-3-shRNA1	5′-CACCGCGGTTACTGCAATGTGGTCATTCAAGAGATGACCACATTGCAGTAACCGCTTTTTTG-3′
	5′-GATCCAAAAAAGCGGTTACTGCAATGTGGTCATCTCTTGAATGACCACATTGCAGTAACCGC-3′
GPC-3-shRNA2	5′-CACCGGCTCTGAATCTTGGAATTGATTCAAGAGATCAATTCCAAGATTCAGAGCCTTTTTTG-3′
	5′-GATCCAAAAAAGGCTCTGAATCTTGGAATTGATCTCTTGAATCAATTCCAAGATTCAGAGCC-3′
GPC-3-shRNA3	5′-CACCGCCGAATGCTCACCAGAATGTTTCAAGAGAACATTCTGGTGAGCATTCGGCTTTTTTG-3′
	5′-GATCCAAAAAAGCCGAATGCTCACCAGAATGTTCTCTTGAAACATTCTGGTGAGCATTCGGC-3′
GPC-3-shRNA4	5′-CACCGAGCAAGACGTGACCTGAAAGTTCAAGAGACTTTCAGGTCACGTCTTGCTCTTTTTTG-3′
	5′-GATCCAAAAAAGAGCAAGACGTGACCTGAAAGTCTCTTGAACTTTCAGGTCACGTCTTGCTC-3′
Negative-shRNA	5′-CACCGTTCTCCGAACGTGTCACGTCAAGAGATTACGTGACACGTTCGGAGAATTTTTTG-3′
	5′-GATCCAAAAAATTCTCCGAACGTGTCACGTAATCTCTTGACGTGACACGTTCGGAGAAC-3′

Complete culture medium with serum and antibiotics is freshly added to each well 60 min before transfection. Dilute 2 μg of DNA and 6 μL of GenJet™ reagent were added into 200 μL of serum-free DMEM with high glucose. Then, this mixture was vortexed gently and spin down briefly to bring drops onto each well. The mixture was homogenized by gently swirling the plate and was incubated at 37 °C. At 12 h posttransfection, the DNA/GenJet™ complex-containing medium was removed and replaced with a completely fresh serum-containing medium, and a second identical transfection was then carried out 24 h later. After the first transfection at 48 h, GPC-3 mRNA or protein levels in the harvested cells were analyzed by fluorescence quantitative reverse transcriptase-polymerase chain reaction (FQ-RT-PCR) and Western blotting, respectively. The transfection cells were diluted (1:10) and stably transfected in a medium containing G418 (400 μg/mL) for 2 weeks and maintained with 200 μg/mL for 3 weeks; the individual clone was isolated and expanded.

### RNA extraction

Total RNA was extracted using TRIzol reagent (Invitrogen, USA) by following the manufacturer’s instructions. Briefly, after HepG2 cells were transfected with or without shRNA at 48 h, the cells (1 × 10^6^) were harvested and washed twice with cold phosphate-buffered saline (PBS). For each well, 1.0 mL TRIzol reagent was added; then, RNA was precipitated by isopropanol, washed with 75 % ethanol, dissolved with 20 μL DEPC (0.1 %), and quantified using a UV spectrophotometer.

### FQ-RT-PCR

Reverse transcription was carried out by using the RevertAid^TM^ First Strand cDNA Synthesis Kit (MBI Fermentas, Vilnius, Lithuania) according to the standard protocol. In brief, in a 20-μL reaction mixture containing 2 μg RNA and oligo primers, at 42 °C for 1 h, cDNA was synthesized by PCR using SYBR®Premix Ex TaqTMII (TaKaRa, Dalian, China) with primers. The primers were GPC-3 forward: 5′-CGAGATAAGCACCTTTCACAACC-3′, GPC-3 reverse: 5′-AGAAGAAGCACACCACCGAGA-3′ (NM_004484), and GAPDH forward: 5′-CAAGGTCATCCATGACAACTTTG-3′and reverse: 5′-GTCCACCACCCTGTTGCTGTAG-3′ (NM_017008). PCR was performed as follows: 30 s of predegeneration at 95 °C, 5 s at 95 °C, and 45 s at 60 °C for 40 cycles with Rotor Gene 3000 thermal cycling instrument (QIAGEN, Valencia, CA). GPC-3 gene and GAPDH gene were amplified in the same reaction as an internal loading control. The amplification specificity was confirmed by the melting curves, the fluorescence was collected at 60 °C (*n* = 5), and the relative quantitative results were analyzed by the 2^−∆Ct^ values.

### Protein extraction

The transfected cells were harvested and washed with cold PBS twice and then lysed on ice in RIPA buffer (1× PBS, 1 % NP-40, 0.1 % sodium dodecylsulfate (SDS), 5 mM EDTA, 0.5 % sodium deoxycholate, and 1 mM sodium orthovanadate) that contained 100 μg/mL phenylmethylsulfonyl fluoride and protease inhibitors (KeyGen, Nanjing, China).

### Western blotting

Approximately 50 μg of protein from each sample was separated using a 10 % SDS–polyacrylamide gel, and it was electrotransferred to polyvinylidene fluoride membranes and blocked in 5 % nonfat dry milk in Tris-buffered saline, pH 7.5 (100 mM NaCl, 50 mM Tris, and 0.1 % Tween-20). The transferred membranes were incubated with anti-GPC-3 (Abcam, USA) and anti-β-actin primary antibodies (Beyotime, China) overnight at 4 °C, followed by incubation with horseradish peroxidase-conjugated rabbit/mouse anti-goat secondary antibodies. Proteins were detected by Quantity One software (Bio-Rad, Laboratories, Inc., USA) using Immobilon ECL Chemiluminescence HRP Substrate (Millipore, Merck, USA).

### Cell proliferation

After transfection for 48 h, cells were seeded in 96-well plates with optical density to adapt to the different time points. Cell proliferation was analyzed by an 3-(4,5-dimethylthiazol-2-yl)-2,5-diphenyltetrazolium bromide (MTT) assay according to the manufacturer’s instruction (Beyotime, China). In brief, after cells were seeded for 4 h, 10 μL of MTT was added into the medium (final, 0.5 mg/mL) and the wells were incubated for 4 h at 37 °C to allow formation of dark blue formazan crystal. The medium was carefully removed with a pipette and 100 μL of dimethyl sulfoxide was added. The mixture was shaken gently and was equilibrated to room temperature to detect absorbance (*n* = 5) at 570 nm with Synergy HT Multi-Detection Microplate Reader (Bio-Tek, USA).

### Cell cycles

HepG2 cells were infected with pGPU6/GFP/Neo-GPC-3 shRNA vector for 72 h. The cells with or without transfected vector were harvested, trypsinized, washed with PBS, and fixed in 70 % precooled ethanol for 24 h at 4 °C. Fixed cells were stained with propidium iodide with RNase A cocktail at 37 ° for 30 min. Data were obtained using FACSCalibur flow cytometer (BD Biosciences, USA).

### Cell apoptosis

The effective apoptosis of transfected cells were evaluated by the PE-labeled Annexin-V/7-AAD assay and DNA-ladder electrophoresis according to the manufacturer’s protocol. In brief, HepG2 cells (2 × 10^5^) were collected, rinsed twice in cold PBS, and added with binding buffer; then, apoptosis rate was analyzed. By DNA-ladder electrophoresis, the apoptosis level was verified as follows: the cells (1 × 10^6^) were trypsinized, added with RNase A and protease K, lysed at 70 °C for 10 min, extracted DNA from the mixture by purification pillar, electrophoresized on 2 % agarose gel, and then analyzed by an ultraviolet transilluminator with Quantity One software (Bio-Rad, Laboratories, Inc., USA).

### Statistical analysis

Data were presented as mean ± standard deviation (SD) and subjected to one-way analysis of variance. Differences between groups were examined by Student’s *t* test unless otherwise noted. Statistical significance was accepted at the level of *P* value less than 0.05 by using SPSS 17.0 software.

## Results

### Efficiency of GPC-3-shRNA plasmid screening

**Fig. 1:**
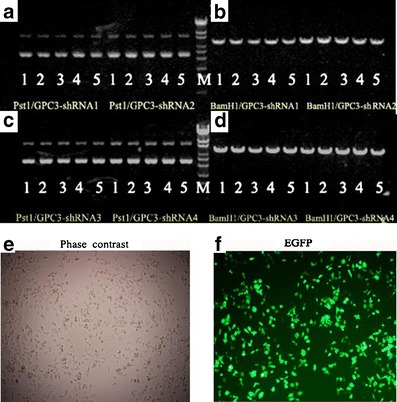
Construction, enzymatic digestion of plasmids, and shRNA transfection efficiency. GPC-3-shRNAs were inserted into pGPU6/GFP/Neo vector and constructed containing shRNA plasmids. The shRNA plasmids were confirmed by enzyme digestion. **a**, **c** Pst I and **b**, **d**, BamH I. HepG2 cells were transfected according to the preoptimized instructions as described in “Materials and methods.” At 24 h, after the HepG2 cells were transfected with different shRNAs, the cells were then harvested and checked. The phase-contrast (**e**) and fluorescence (**f**) photomicrographs (×100 magnification) of the cells transfected with different constructed shRNA plasmids with or without green fluorescent protein at 24 h

The different kinds of GPC-3shRNAs were synthesized according to the GPC-3 sequence including the negative shRNA (Table 1), were inserted into pGPU6/GFP/Neo vectors, and constructed containing shRNA plasmids. All the inserted sequences, confirmed by sequencing or by endonuclease BamH I and Pst I enzymatic digestion, and efficiency of shRNA transfection are shown in Fig. 1. The inserted fragments in plasmids verified the constructed plasmids correctly and successfully for transfection with a transfection reagent (Fig. 1a–d). HepG2 cells were transfected with the following plasmids: pGPU6/GFP/NeoGPC-3shRNA1 (shRNA1), pGPU6/GFP/NeoGPC-3 shRNA2 (shRNA2), pGPU6/GFP/NeoGPC-3shRNA3 (shRNA3), and pGPU6/GFP/ NeoGPC-3shRNA4 (shRNA4). The cells with a higher efficiency (over 80 %) at 24 h were observed under selected visual fields (*n* = 5/each) at random with an inverted fluorescence microscope for counting transfected cells (Fig. 1b, 1f).

### Downregulation of GPC-3 expression by shRNA transfection

**Fig. 2:**
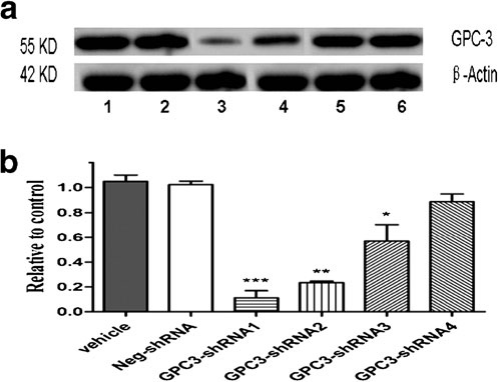
Suppression of GPC-3 expression after HepG2 cells were transfected with different shRNAs. **a** After HepG2 cells were transfected with different shRNAs at 24 h, the alterations of GPC-3 expression at protein level were analyzed by Western blotting as described in “Materials and methods.” *Lane 1*, the control (the untreated cells, vehicle), *2* the neg-shRNA, *3* the shRNA1, *4* the shRNA2, *5* the shRNA3, and *6* the shRNA4. **b** The ratio of GPC-3 protein expression. Data were expressed as mean ± SD from three independent experiments. *P < 0.05, **P < 0.01, and ***P < 0.001 vs. the neg-shRNA group

After HepG2 cells were transfected with different shRNAs at 24 h, the suppression of GPC-3 expressions at gene transcriptional level analyzed by FQ-RT-PCR is shown in Table 2. Different silencing efficiencies were observed after the cells were transfected with shRNAs, and the inhibited rate of GPC-3 mRNA was 90.4 % in shRNA1, 77.6 % in shRNA2, 46.8 % in shRNA3, and 15.3 % in shRNA4. No significant difference exists between the negative-shRNA (neg-shRNA) group and the control group. shRNA1 was one of the best plasmids with high efficiency (*t* = 21.786, *P* < 0.001). The same interference effects were observed in shRNA1 (*P* < 0.001), shRNA2 (*P* < 0.01), and shRAN3 (*P* < 0.05), and no statistical difference in the shRNA4 was observed. The different inhibition efficiencies at protein level were evaluated by Western blotting (Fig. 2a). shRNA1 was one of the best specific plasmids for GPC-3 (Fig. 2b), indicating that the highly specific and efficient shRNA1 could suppress the GPC-3 activation in hepatoma cells at the gene transcriptional or protein level.

**Table 2:** Relative quantitative analysis of GPC-3 mRNA expression (mean ± SD) after HepG2 cells were transfected with different shRNAs (*n* = 5)

Group	mRNA (2^−∆Ct^)	Inhibited (%)^a^	*t* value	*P* value
Control	1.05 ± 0.05	0	–	–
Negative-shRNA	1.02 ± 0.04^b^	2.86	0.781	0.984
GPC-3-shRNA1	0.11 ± 0.06^b^	90.4	21.786	<0.001
GPC-3-shRNA2	0.23 ± 0.12^b^	77.6	28.921	<0.001
GPC-3-shRNA3	0.57 ± 0.13^b^	46.8	5.873	<0.001
GPC-3-shRNA4	0.89 ± 0.06^b^	15.3	3.691	0.054

*mRNA* GPC-3 mRNA level, *Inhibited (%)* the inhibited percentage, *P values* the data were calculated by Dunnett’s *t* method

^a^The inhibited (%) = 1 − 2^−∆∆Ct^ from ∆Ct = Ct_GPC-3_ − Ct_GAPDH_ and ∆∆Ct = ∆Ct_Sample_ − ∆Ct_Control_

^b^Compared with the control group

### Suppression of cell proliferation by shRNA1

**Fig. 3:**
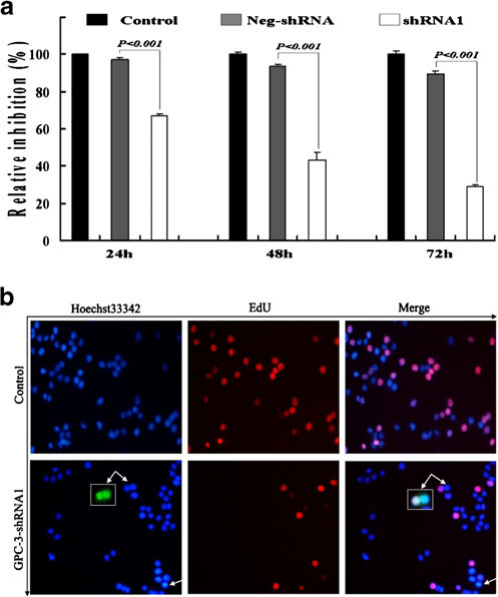
The effect of silencing GPC-3 on HepG2 cell growth and survival. **a** The downregulation of GPC-3 expression inhibited the HepG2 cell viability with a time-dependent manner. Data represented the mean ± SD from three independent experiments. **b** After the HepG2 cells (×200) were transfected with shRNA and stained with EdU and Hoechst 33342. At 48 h, the cells were plated in 96-well plates (2.0 × 10^3^cells/well) in triplicate for another 24 h, then exposed to EdU for 2 h, and visualized under a fluorescence microscope. EGFP (*green*) labeled by EdU was shown with *double white arrows*, and the *single white arrow* showed that the cells had been induced to apoptosis after silencing GPC-3 by shRNA1. EdU (*red*), DNA synthesis; Hoechst 33342 (*blue*), nuclear staining; apoptotic cells (*white arrows*) showed shrunken nuclei with a bright fluorescent appearance

After HepG2 cells were transfected with shRNA1, the effective and significant inhibition of cell proliferation is shown in Fig. 3. Compared with the neg-shRNA group, the viability after the cells transfected with shRNA1 was notably inhibited in a time-dependent manner. The higher death rates of the transfected cells were found significantly in the shRNA1 group compared with the neg-shRNA group at 24 h (33.2 vs. 2.86 %, *P* < 0.001), 48 h (57.0 vs. 6.5 %, *P* < 0.001), and 72 h (71.1 vs. 10.5 %, *P* < 0.001, Fig. 3a), whereas the neg-shRNA group showed a similar frequency of death as the control group. DNA synthesis and nuclear staining in the transfected cells stained with 5-ethynyl-2′-deoxyuridine (EdU) or Hoechst 33342 were visualized under a fluorescence microscope (Fig. 3b), with apoptotic cells showing shrunken nuclei with a bright fluorescence appearance, indicating that high GPC-3 expression increases the growth and survival of HepG2 cells in vitro.

### Synergistic effect of shRNA1 plus sorafenib

After HepG2 cells were transfected with shRNA1 for 48 h, different doses of sorafenib (from 0 to 100 μM/L) were simultaneously added into the cells and were cocultured for 24 h; the synergistic effects of shRNA1 plus sorafenib on cell growth evaluated by an MTT assay are shown in Table 3. GPC-3 expression in HepG2 cell was downregulated notably by shRNA in a dose-dependent manner. The combination of shRNA1 plus sorafenib has showed higher inhibition effects (*P* < 0.001) on cell proliferation, which demonstrated that the synergistic effect on cell proliferation can be inhibited after the cells were transfected with shRNA1 plus sorafenib.

**Table 3:** Synergistic effect of HepG2 cell proliferation after shRNA1 transfection with different doses of sorafenib (*n* = 5)

Group	Control	neg-shRNA	shRNA1 + sorafenib	*t* value	*P* value
0 μM	1.23 ± 0.05	1.22 ± 0.03	0.96 ± 0.24^a^	2.147	0.040
2.5 μM	1.20 ± 0.05	1.17 ± 0.02	0.73 ± 0.03^a^	14.972	<0.001
5 μM	1.15 ± 0.04	1.16 ± 0.02	0.68 ± 0.04^a^	15.805	<0.001
10 μM	1.08 ± 0.03	1.05 ± 0.08	0.54 ± 0.03^a^	23.314	<0.001
20 μM	1.02 ± 0.03	1.00 ± 0.03	0.51 ± 0.03^a^	18.713	<0.001
50 μM	0.96 ± 0.05	0.94 ± 0.07	0.36 ± 0.03^a^	19.221	<0.001
100 μM	0.86 ± 0.01	0.86 ± 0.06	0.17 ± 0.02^a^	50.352	<0.001

*neg-shRNA* the negative shRNA, *shRNA1* the GPC-3-shRNA1

*Compared with the control group

### Alteration of HepG2 cell cycles after shRNA1 transfection

**Fig. 4:**
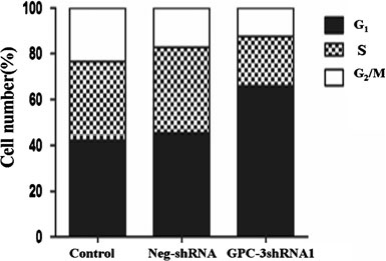
Alteration of cell cycles after HepG2 cells were transfected with shRNA1. The cell cycle progression was inhibited through G_1_ arrest and increased up to 35.98 % compared with the control group (*t* = −6.382, *P* = 0.024). Apoptotic cells were examined after cells stained with Annexin V-PE/7-AAD were analyzed by flow cytometry

After HepG2 cells were transfected with shRNA1, the alteration of cell cycles by FACS analysis of PI-stained cells is shown in Fig. 4. The portion of G_1_ phase cells was increased by approximately 35.98 % (*t* = −6.382, *P* = 0.024), and the portion of G_2_/M phase cells was decreased by a comparable degree. The stable silencing of GPC-3 dramatically suppressed the HepG2 cells’ cycle progression and arrested in G_1_ phase, which indicated that silencing of GPC-3 gene could inhibit effectively the proliferation and growth of HepG2 cells by G_1_ phase arrest.

### Increase in HepG2 cell apoptosis after shRNA1 transfection

**Fig. 5:**
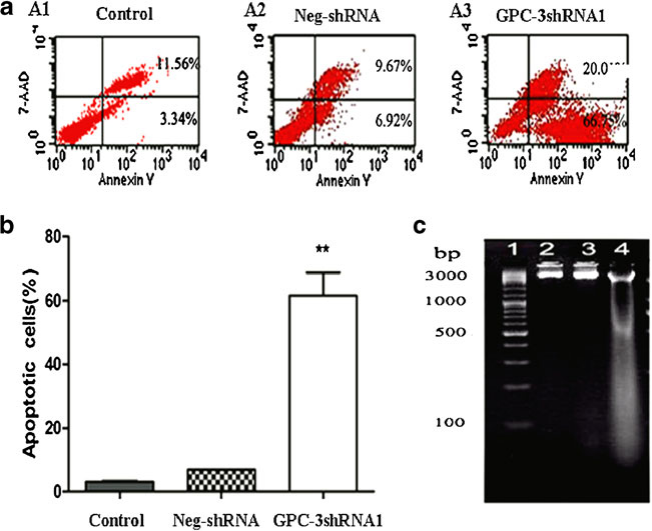
Apoptosis increased after HepG2 cells were transfected with shRNA1. **a** The number of apoptotic cells of the knockdown group was significantly higher than the negative or control group. **b** The number of cells with nuclear morphological features of apoptosis was counted after staining with Hoechst 33342. Data are expressed as percentage of apoptotic cells based on counting 100 cells in randomly selected fields. Data are from three separate experiments (mean ± SD). **c** DNA was extracted and DNA-ladder electrophoresis assay was used to detect apoptosis. *1*, DNA marker; *2*, the control group; *3*, the negative-shRNA; and *4*, the shRNA1 group. When the cells were apoptotic, the DNA was degraded and the DNA ladder would be detected on agarose gel electrophoresis. **P < 0.01

The potential effects of shRNA-mediated GPC-3 silencing on cell apoptosis with an Annexin V-PE/7-AAD assay by flow cytometry are shown in Fig. 5. Compared with the control (3.34 %, Fig. 5, A1) or negative-shRNA (6.92 %, Fig. 5, A2) group, the number of apoptotic HepG2 cells was significantly increased in the shRNA1 group (66.75 %, Fig. 5, A3). The apoptosis rate of HepG2 cells was increased significantly (Fig. 5b, *t* = −11.456, *P =* 0.008). In order to further evaluate the effects of shRNA1 transfection on cell apoptosis, the DNA degradation from HepG2 cells was observed obviously on DNA-ladder electrophoresis (Fig. 5c) in the shRNA1 group, whereas this was not detected in the negative or control group, suggesting that the inhibition of GPC-3 can remarkably induced apoptosis of HepG2 cells.

## Discussion

HCC is a common malignant disease with poor prognosis [19, 20]. Its underlying molecular mechanism provides a potential in developing new therapeutic target, which is very urgent [21, 22]. GPC-3 is a 66-kDa protein, which belongs to the family of heparan sulfate proteoglycans, bound to the cell surface by a glycosylphosphatidylinositol anchor. It is significantly overexpressed in HCC, embryo tissues, fetal liver, and trophoblastic cells and has little or no expression in adult tissues except for normal lung, ovarian, mammary epithelial, and mesothelial tissues [23, 24]. Previous studies have shown that GPC-3 as an oncofetal protein was abnormally expressed during HCC development in human or animal models [17]. In this study, specific and efficient shRNA plasmids were constructed to explore the effect of GPC-3 downregulation on the growth, as well as apoptosis-related mechanisms, of hepatoma cells, and it was found out that they inhibited successfully and effectively the GPC-3 expression of human HepG2 cells.

GPC-3 is essential for proper embryogenesis and represents a promising target structure [25, 26]. Variant approaches for the functional characterization of GPC-3 in different cell types have been developed [27, 28]. However, these strategies cannot have specificity, efficiency, stability, and nontoxicity at the same time. A developing RNAi strategy is a powerful technique to inhibit specific gene expression, exhibit the potential to study gene function, or explore new tumor therapeutic agents [29, 30]. In the present study, four kinds of GPC-3-targeting shRNA expression vectors were applied (pGPU6/GFP/NeoGPC-3shRNA1, 2, 3, and 4) to target GPC-3 gene sites. The most significant inhibition of GPC-3 expression at RNA level was that of the shRNA1 groups, indicating that special shRNA application could be an effective GPC-3 gene-silencing method. Further studies revealed that the level of GPC-3 gene and its expression was significantly decreased in the shRNA1-transfected cells compared with untreated and control cell groups, suggesting that shRNA1 not only intervened the expression of GPC-3 gene but also inhibited the action of transcription.

shRNA1 downregulated GPC-3 expression to suppress tumor cell proliferation and had a synergistic effect with chemotherapy drug. After HepG2 cells were transfected with shRNA1, the effects of downregulated GPC-3 expression on cell proliferation were investigated in the present study. GPC-3-shRNA1 remarkably downregulated the GPC-3 expression of HepG2 cells at the mRNA or protein level, with 71.1 % of cell proliferation suppression at 72 h; the cell cycle arrested at G_1_ phase and notably induced the HepG2 cell apoptosis. To further evaluate the effect of shRNA and chemotherapy drug on HepG2 cells, different doses of sorafenib were added into the transfected cells, and the result demonstrated that shRNA and sorafenib have a synergistic effect on the inhibition of HepG2 cell proliferation, suggesting that knockdown GPC-3 gene could effectively and significantly suppress the proliferation and induce apoptosis, and confirming that GPC-3 is a potential effective target for HCC therapy [31, 32].

The molecular biology of carcinogenesis and tumor progression of HCC has been increasingly understood with intense research in recent years [33, 34]. The heterogeneity of HCC molecular pathology poses a formidable obstacle to the development of noncytotoxic therapies. Several protumorigenic signaling pathways can be aberrantly activated in HCC, including those triggered by Wnts. GPC-3, a membrane-bound heparan sulfate proteoglycan that is overexpressed in most HCCs, promotes the tumor growth by stimulating Wnt signaling, because GPC-3 binds with high affinity to Wnts and its growth-promoting activity requires attachment to the cell membrane [13, 14]. The role of several growth factors and angiogenic factors has been confirmed. Effective agents targeting these molecular abnormalities have been developed and widely tested in preclinical studies of HCC cell lines or xenograft models [25].

In summary, the management of advanced HCC is entering the era of molecular targeting therapy [35, 36], which is of particular significance for HCC in view of the lack of existing effectively systemic therapy for HCC. The transcription and activation of intervened GPC-3 gene by specific shRNA expression of vector mediated RNAi were successfully used to suppress the GPC-3 expression in human HepG2 cells with the viable alteration, proliferation inhibition, and apoptosis occurrence of hepatoma cells. The present data made it clear that GPC-3 plays an important role in HCC, and it should be a promising and potential molecular therapeutic target for HCC to improve prognosis.

## Abbreviations

EdU: 5-Ethynyl-2′-deoxyuridine
EGF: Epidermal growth factor
FQ-RT-PCR: Fluorescence quantitative reverse transcriptase-polymerase chain reaction
GPC-3: Glypican-3
HCC: Hepatocellular carcinoma
PBS: Phosphate-buffered saline
SD: Standard deviation
shRNA: Short hairpin RNA
VEGF: Vascular endothelial growth factor
